# Glycyrrhizin as a Nitric Oxide Regulator in Cancer Chemotherapy

**DOI:** 10.3390/cancers13225762

**Published:** 2021-11-17

**Authors:** Minsu Kim, Seok Chan Park, Dong Yun Lee

**Affiliations:** 1 Department of Bioengineering, College of Engineering, Hanyang University, Seoul 04763, Korea; kms06@naver.com (M.K.); randypark100@gmail.com (S.C.P.); 2 Institute of Nano Science & Technology (INST), Hanyang University, Seoul 04763, Korea; 3 Elixir Pharmatech Inc., Seoul 04763, Korea

**Keywords:** glycyrrhizin, multidrug resistance, nitric oxide, chemotherapy, tumor microenvironment, ATP-biding cassette

## Abstract

**Simple Summary:**

Glycyrrhizin (GL) has anti-cancer, anti-inflammatory, anti-viral, and anti-oxidant activity. In particular, GL reduces multidrug resistance (MDR) in cancer cells, which is a major obstacle to chemotherapy. Nitric oxide (NO) also plays an important role in MDR, and GL affects NO concentration in the tumor microenvironment. However, the effects of GL and NO interaction on MDR have not been reviewed. Here, we review the role of GL as an NO regulator in cancer cells and its subsequent anti-MDR effect and posit that GL is a promising MDR inhibitor for cancer chemotherapy.

**Abstract:**

Chemotherapy is used widely for cancer treatment; however, the evolution of multidrug resistance (MDR) in many patients limits the therapeutic benefits of chemotherapy. It is important to overcome MDR for enhanced chemotherapy. ATP-dependent efflux of drugs out of cells is the main mechanism of MDR. Recent studies have suggested that nitric oxide (NO) can be used to overcome MDR by inhibiting the ATPase function of ATP-dependent pumps. Several attempts have been made to deliver NO to the tumor microenvironment (TME), however there are limitations in delivery. Glycyrrhizin (GL), an active compound of licorice, has been reported to both reduce the MDR effect by inhibiting ATP-dependent pumps and function as a regulator of NO production in the TME. In this review, we describe the potential role of GL as an NO regulator and MDR inhibitor that efficiently reduces the MDR effect in cancer chemotherapy.

## 1. Introduction

Cancer is a leading cause of death worldwide, accounting for nearly 10 million deaths in 2020. Among the methods that have been developed to treat cancer, chemotherapy is used primarily to treat metastatic cancer that cannot be eradicated with surgery or radiotherapy. Although chemotherapy is the most effective method to eradicate cancer, it involves several challenges such as evolution of multidrug resistance (MDR) in cancer cells, which includes altered drug activation due to metabolism and excretion, and increased repair of DNA damage caused by anti-cancer drugs. The major mechanism underlying MDR is efflux of drugs at the cell membrane by ATP-binding cassette (ABC) transporters [1,2,3]. Generally, cancer cells overexpress ABC transporters, such as P-glycoprotein (P-gp), multidrug resistance-associated protein 1 (MRP1), and breast cancer resistance protein (BCRP), which limit drug accumulation in the cell. Therefore, inhibiting ABC transporters can be an effective way for sensitizing the cancer cells to chemotherapeutic drugs [4,5,6,7].

Several studies have reported attempts to reverse the MDR effect in cancer cells using various molecules, including nitric oxide (NO). NO is a free radical that plays a biphasic role in cancer cells depending on its concentration. Low concentrations of NO promote cancer cell proliferation and progression by the Warburg effect, while higher concentrations of NO induce DNA damage and apoptosis in cancer cells by activating the apoptosis signal-regulating kinase 1 (ASK1)/c-Jun N-terminal protein kinase (JNK1), BCL-2-associated X protein (BAX), and BCL-2 homologous antagonist killer (BAK) pathways [8,9,10]. Researchers have attempted to deliver high concentrations of NO directly or through NO donors for cancer treatment. Results indicate that NO inhibits not only the MDR effect by hindering ATPase activity, but also cancer cell growth [11,12,13]. However, NO delivery is limited by its toxicity and difficulty in achieving optimal concentration [14].

Glycyrrhizin (GL), isolated from *Glycyrrhiza glabra* (licorice) root, has anti-cancer, anti-inflammatory, and anti-oxidant activities. GL enhances NO production from macrophages stimulated with interferon-gamma (IFN-γ) or lipopolysaccharide (LPS), and the resulting high concentration of NO products kills cancer cells [15,16]. GL upregulates inducible NO synthase (iNOS) by activating the nuclear factor kappa B (NFκB) signaling pathway in macrophages [17]. Furthermore, as mentioned above, high concentration of NO inhibits ABC transporters, which are responsible for MDR in cancer [13,18]. GL also has an MDR reduction effect by itself, increasing cellular uptake through opening of tight junctions and inhibiting efflux of drugs from cancer cells [19,20]. In this review, we describe GL as a prospective MDR inhibitor in cancer chemotherapy that exerts its MDR protective effects by inhibiting ABC transporters through NO regulation. Use of GL in chemotherapy would be an effective, non-toxic approach to increasing NO availability in the cancer microenvironment compared to direct delivery of NO or NO donors.

## 2. Multidrug Resistance in Cancer Chemotherapy

The most common cancer treatment methods are surgery, radiotherapy, and chemotherapy. Each method can be applied alone or in combination based on cancer type, stage, and patient condition. Although surgery often is used as the primary method to treat cancer, additional techniques are required for complete removal of cancer. Radiotherapy, which involves the use of high doses of radiation to remove cancer, can be used to treat regional cancer. However, like surgery, radiotherapy cannot eliminate the possibility of metastatic cancer. Chemotherapy refers to administration of anti-cancer drugs including antimetabolites, genotoxic drugs, and mitosis inhibitors to control proliferation and promote apoptosis of cancer cells. In fact, chemotherapy can be applied to treat various cancer types including metastatic cancer, which is limited in other methods [5,21,22].

### 2.1. Mechanisms of MDR in Chemotherapy

**Figure 1:**
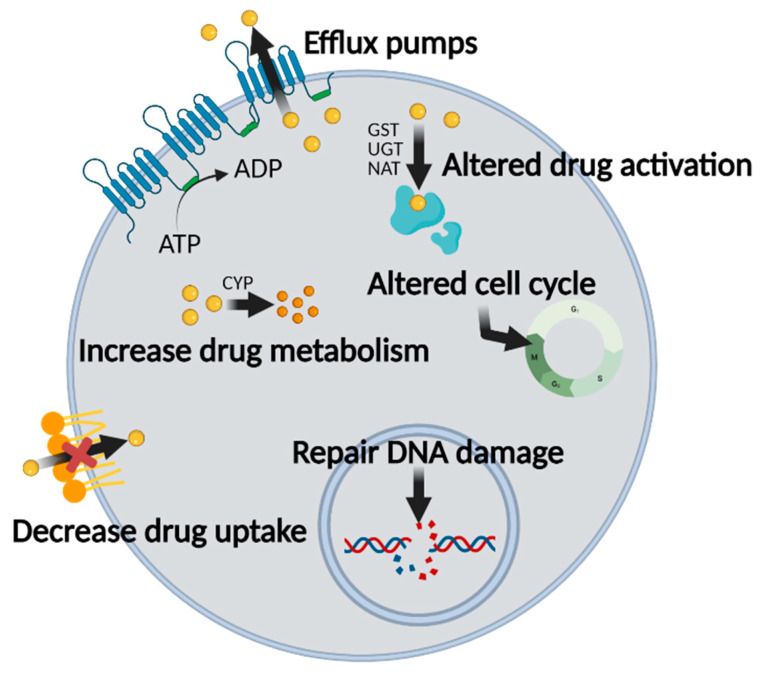
Brief overview of the various mechanisms of MDR that evolve in cancer cells. Image created with BioRender icons. Mechanisms of MDR include efflux via ATPase-dependent membrane pumps; altered drug activation; increased drug metabolism; decreased drug uptake through alterations in membrane lipids; altered cell cycle; and repair of DNA damage to inhibit apoptosis.

There are several obstacles in chemotherapy that decrease its therapeutic effects, including MDR. Types and mechanisms of MDR are shown in Figure 1. Aside from inappropriate drug administration, clinical failure primarily occurs at the cell membrane. Solute carriers (SLCs) in the cell membrane are responsible for mediating the influx of nutrients and drugs. In cancer cells, however, SLC transporters are expressed differently than in normal cells [23,24,25,26]. Therefore, it is important to consider the expression pattern of SLC transporters in chemotherapy. Furthermore, cancer cells have alterations in plasma membrane phospholipid composition and lipid metabolism. Long saturated fatty acid chains, which are associated with increased levels of cholesterol, decrease membrane fluidity to decrease drug uptake [27]. Ceramides, which are long-chain membrane lipids composed of sphingosine and fatty acids, are involved in apoptosis and cell cycle arrest. Glucosylceramide synthase metabolizes ceramides into glucosylceramides (glycosylated form of ceramides) that no longer have pro-apoptotic activity. It has been reported that cancer cells express high levels of glucosylceramide synthase, which decreases the efficacy of apoptosis-inducing and cell cycle-arresting anti-cancer drugs [28,29,30]. Internalized anti-cancer drugs are metabolized by enzymes through phase I and phase II reactions. Phase I reactions include oxidation, reduction, and hydrolysis, which are mediated by cytochrome P450 enzymes (CYPs) and epoxide hydrolases [31,32,33]. Phase II reactions are conjugation reactions that include glucuronidation, sulfation, and glutathionylation. These conjugation reactions are mediated by glutathione S-transferase, arylamine N-acetyltransferases, and sulfotransferases [34,35,36]. As a result, internalized anti-cancer drugs are metabolized or modified, resulting in alteration of their activity [24,37]. Uncontrolled growth and inhibition of apoptosis are the hallmarks of cancer and translate into MDR. Apoptotic pathways in cancer cells are disrupted by downregulation of BAX and BAK or upregulation of anti-apoptotic B-cell lymphoma-2 (BCL-2) proteins [38,39]. Above all, however, the major mechanism of MDR is efflux of drugs against a concentration gradient via ABC transporters in an ATP-dependent manner [2,3,40]. Each of these MDR mechanisms decreases the therapeutic effects of anti-cancer drugs.

### 2.2. ABC Transporters Are Involved in MDR

ABC transporters make a significant contribution to MDR by functioning as efflux pumps. There are 49 ABC genes classified into seven subfamilies in the human genome. ABC family members play important roles in cellular processes such as eliminating waste, signaling, and mediating homeostasis. According to recent studies, 12 ABC transporters are responsible for drug efflux. Among them, ABCB1 (P-gp), ABCC1 (MRP1), and ABCG2 (BCRP) are the main transporters involved in MDR [41,42,43].

P-gp (known as MDR1 or ABCB1) is the first discovered ABC family transporter and acts as a major mediator of MDR in cancer cells. It is present in cells that are involved in absorption, distribution, and elimination such as gastrointestinal tract, liver, and kidney cells. P-gp has broad substrate specificity and recognizes hydrophobic natural products, antibiotics, peptides, and steroids, among other molecules [41,44]. Binding of substrate to P-gp activates the ATP-binding domain, and the subsequent ATP hydrolysis leads to a conformational change in P-gp that results in efflux of substrates [45]. Another ABC transporter, MRP1 (known as ABCC1), was cloned originally from lung cancer cells but is expressed widely in most cancer cells. Like P-gp, MRP1 is involved in efflux of hydrophobic substrates, but unlike P-gp, MRP1 transports anion products conjugated with glucoronate and sulfate [46,47]. After the discovery of MRP1, researchers found many other ABC transporters in various cancer cells. BCRP (known as ABCG2) was identified first in breast cancer cells but is mainly expressed in liver, intestine, and brain cells. As BCRP has a wide range of substrate types, it functions physiologically as a defense system in cancer cells, contributing to MDR [48,49]. 

### 2.3. Strategies to Overcome MDR

One approach to overcoming MDR in cancer chemotherapy is inhibition of ABC transporter activity. RNA interference (RNAi) or small interfering ribonucleic acid (siRNA) has been used to silence ABC transporter gene expression. RNAi and siRNA have been introduced into cells after encapsulation with nanoparticles or by short hairpin RNA transfection to silence P-gp or BCRP genes, resulting in reduced MDR [50,51,52,53,54]. Use of ABC transporter inhibitors can inhibit MDR. First-generation MDR inhibitors (e.g., verapamil, quinine, and cyclosporine A) act as competitive antagonists of ABC transporters but have toxic side-effects. Some second-generation MDR inhibitors (e.g., PSC-833 and VX-710) are less toxic than first-generation inhibitors, but the pharmacokinetics of these drugs requires further optimization. Current second-generation MDR inhibitors (e.g., zosuquidar, elacridar, and tariquidar) show fewer pharmacokinetic interactions than PSC-833 and VX-710 due to limited interactions with cytochrome P450 family 3 and subfamily A (CYP3A) proteins [7,55,56,57]. Many studies have aimed to increase the efficacy of anti-cancer drugs by delivering MDR inhibitors and anti-cancer drugs together, as shown in Table 1. A recent study showed that MDE can be overcome by ATPase inhibition, which is necessary for ABC transporter activity. NO delivered directly or by NO donors inhibits not only MDR by inhibiting ATPase activity, but also cancer cell growth [13,14,58].

#### 2.3.1. P-gp Inhibitors

Although no P-gp inhibitors have been approved for clinical use, P-gp inhibitors show significant efficacy in reducing MDR and increasing the therapeutic effects of chemotherapy drugs in vitro and in vivo [67]. P-gp inhibitors are usually loaded into nanoparticles with anti-cancer drugs. For example, Bajelan et al. encapsulated PSC-833 (a second-generation MDR inhibitor) into nanoliposomes with doxorubicin (DOX) and then used these nanoliposomes to treat breast cancer cells. Co-encapsulation of DOX and PSC-833 reduced MDR, resulting in a potent anti-cancer effect [59]. Another approach for P-gp inhibition is related to cyclooxygenase-2 (Cox-2). Cox-2 is expressed highly in a wide range of cancer cell types, and Cox-2 overexpressing cells also exhibit increased P-gp activity. When renal mesangial cells were treated with the Cox-2 inhibitor NS398, P-gp activity was blocked and MDR effects were decreased. These results suggest a link between Cox-2 and P-gp-mediated MDR [75]. Rahman et al. examined the potential of Cox-2 inhibition in colon cancer. They combined 5-fluorouracil as a chemotherapy agent and celecoxib as a Cox-2 inhibitor to treat colorectal cancer. They demonstrated an increase in chemosensitivity through inhibition of P-gp and suggested that combing Cox-2 inhibitors with drugs has potential benefits in chemotherapy [76].

#### 2.3.2. MRP1 Inhibitors

Most cells express MRP1, but MRP1 is expressed at very high levels in the blood–brain barrier. Thus, chemotherapy to treat brain cancer is limited by MDR [77,78]. Reversan is a non-toxic selective inhibitor of MRP1 that has been evaluated in vitro and in vivo. Tivnan et al. treated glioblastoma multiforme (GBM) through co-delivery of temozolomide and Reversan. Co-delivery of temozolomide and Reversan noticeably decreased the viability of patient-derived GBM cells (A172 and U251) compared to temozolomide treatment only [73]. Kopanitsa et al. combined Reversan with anti-cancer agents (phosphodiesterase inhibitors such as PF-2545920, PQ10, and papaverine) and reported higher suppression of GBM cell viability than with treatment of the anti-cancer agents alone [74].

#### 2.3.3. BCRP Inhibitors

BCRP has substrate specificity for tyrosine kinase inhibitors. Fumitremorgin C (FTC) is one of the most effective competitive inhibitors for BCRP binding. Gefinitib is used widely for breast cancer treatment. Liu et al. used gefinitib and FTC to treat human breast cancer cells (MCF-7) and found that FTC significantly increased the inhibitory effect of gefinitib on MCF-7 cell growth [66]. Wang et al. used polymeric micelle-based doxorubicin and lapatinib treatment to address MDR in breast cancer cells. Lapatinib binds to the ATP binding sites of BCRP and inhibits MDR. The combination of doxorubicin and lapatinib showed potential for preventing MDR in breast cancer cells [68]. Pluronic block copolymers arranged in a triblock structure with poly(ethylene oxide) (PEO) blocks and poly(propylene oxide) (PPO) blocks have been shown to inhibit the activity of P-gp and BCRP. Wei et al. used paclitaxel-loaded Pluronic P123/F127 mixed micelles for lung cancer treatment and found that use of these micelles increased the anti-cancer activity of paclitaxel by overcoming MDR in the cancer cells [69].

## 3. Physiology of Nitric Oxide

### 3.1. Synthesis of Nitric Oxide

**Figure 2:**
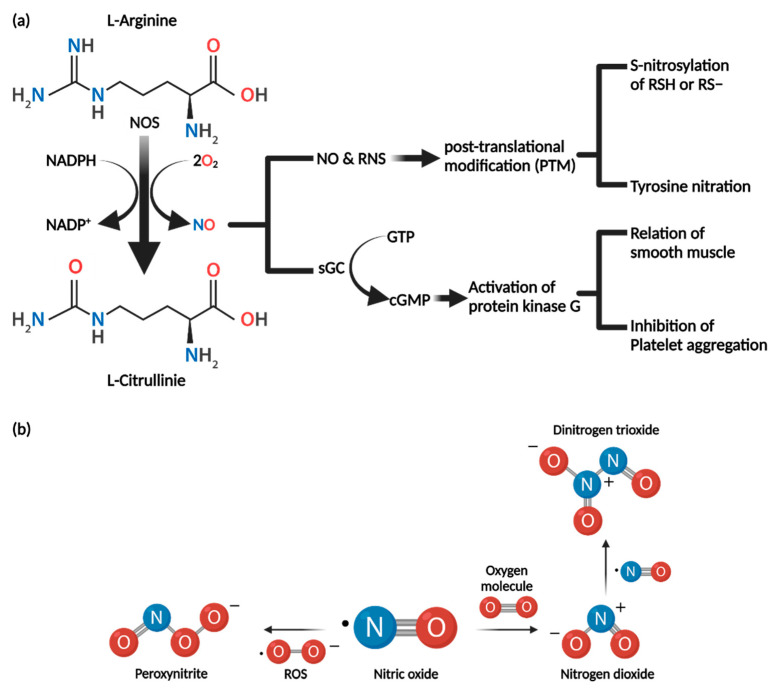
NO mechanism of synthesis and action. (**a**) NO is synthesized in the process of converting L-arginine to L-citrulline and is oxidized by NOS in the presence of O_2_ and NADPH. There are two major mechanisms of action of NO: cGMP dependent and cGMP independent. The NO/cGMP pathway induces relaxation of smooth muscle and inhibits platelet aggregation. In the cGMP independent pathway, some NO is converted into reactive nitrogen species (RNS). NO and RNS mediate post-translational protein modification (PTM) by S-nitrosylation and tyrosine nitration. (**b**) Synthesis of dinitrogen trioxide (N_2_O_3_) and peroxynitrite (ONOO^−^).

NO is synthesized by nitric oxide synthase (NOS)-catalyzed conversion of l-arginine to l-citrulline by oxidation in the presence of O_2_ and NADPH, as briefly shown in Figure 2 [79]. There are three NOS types: NOS1 (neuronal NOS, nNOS), NOS2 (inducible NOS, iNOS), and NOS3 (endothelial NOS, eNOS) [80,81]. eNOS and nNOS isoforms generally are co-expressed in many cell types and are referred to as constitutive NOS (cNOS). By contrast, inducible NOS (iNOS) is transcribed and expressed only in the presence of a specific stimulus such as cytokines. NO synthesis by cNOS is dependent on intracellular calcium concentration (calcium–calmodulin dependence). When the intracellular calcium concentration is increased, the amount of activated Ca^2+^/calmodulin complex increases due to binding of calcium. This induces phosphorylation of protein kinases involved in NOS expression and increases cNOS expression. To summarize, cNOS expression is controlled by negative feedback of intracellular calcium concentration and results in production of small amounts of NO for a short period of time to control nerve (nNOS) and blood vessel function (eNOS). As mentioned above, iNOS is not expressed normally but in response to the presence of external stimuli such as cytokines. Furthermore, expression of iNOS is not calcium dependent; once expressed, large amounts of NO are produced over a long period of time [80,81].

### 3.2. Biochemical Properties of Nitric Oxide

NO is a short-lived free radical with high reactivity that can diffuse easily in cell membranes and acts as an intracellular messenger [82]. Because of its high reactivity, it reacts with biomolecules such as DNA, proteins, and lipids in cells. Through reaction with NO, biomolecules are deactivated or activated [79,82,83]. NO can form other reactive intermediates. As NO has unpaired electrons, it reacts with reactive oxygen species (ROS) to form reactive nitrogen species (RNS) such as dinitrogen trioxide (N_2_O_3_) and peroxynitrite (ONOO^−^) [84,85]. These RNS influence protein function and, therefore, the function of organisms. Dinitrogen trioxide (N_2_O_3_) and peroxynitrite (ONOO^−^) can cause DNA damage [85]. Dinitrogen trioxide (N_2_O_3_) forms N-nitrosamines through nitrosate amines. N-nitrosamines are formed by dinitrogen trioxide alkylating DNA, leading to destabilization and increased breakage of the DNA. Peroxynitrite (ONOO^−^) can oxidize and add nitrate groups to DNA [84]. It can also cause single-stranded DNA breaks through attack of the sugar–phosphate backbone. The biochemical effects of NO depend on several factors. Factors include formation and metabolism of NO, types of NOS present, and most importantly, concentration of nitric oxide present.

### 3.3. Nitric Oxide Mechanism of Action

There are two major mechanisms of action of NO: cyclic GMP (cGMP)-dependent and cGMP-independent [86].

#### 3.3.1. cGMP-Dependent Pathway

Soluble guanylate cyclase (sGC) contains two heme groups to which NO binds. When NO binds to the heme groups of soluble guanylate cyclase (sGC), cGMP is generated by conversion from GTP [87]. cGMP has many effects on cells, mainly mediated by activation of protein kinase G (PKG). PKGs activated by NO/cGMP relax vascular and gastrointestinal smooth muscle and inhibit platelet aggregation [88].

#### 3.3.2. cGMP-Independent Pathway

NO mediates reversible post-translational protein modification (PTM) and signal transduction by S-nitrosylation of cysteine thiol/sulfhydryl residues (RSH or RS^−^) in intracellular proteins. S-nitrosothiol derivatives (RSNO) form as a result of S-nitrosylation of protein. S-nitrosylation influences protein activity, protein–protein interactions, and protein localization [89,90]. S-Nitrosylation upon excessive generation of RNS results in nitrosative stress, which perturbs cellular homeostasis and leads to pathological conditions. Therefore, nitrosylation and de-nitrosylation are important in S-nitrosylation-mediated cellular physiology [89].

Tyrosine nitration results from reaction with peroxynitrite (ONOO^−^), which is an RNS formed by interaction of NO and ROS. Tyrosine nitration covalently adds a nitro group (-NO_2_) to one of the two equivalent ortho carbons of the aromatic ring of tyrosine residues. This affects protein function and structure, resulting in loss of protein activity and changes in the rate of proteolytic degradation [89].

## 4. Nitric Oxide and Cancer

Studies on the effects of NO on cancer formation and growth have been contradictory. There are several reasons for these contradictory findings. These include NO concentration, duration of NO exposure, sites of NO production, type of NOS, sensitivity of the experimental tissue to NO, and whether peroxide is produced [91]. Cancer tissue contains not only cancer cells, but also immune cells. In cancer tissues, NO is produced mainly by iNOS and expressed in macrophages and cancer cells, and small amounts of eNOS and nNOS are produced [92]. When NO is produced in cancer tissues, the promotion or inhibition of cancer growth can depend on the relative sensitivities of given cancer cells and immune cells to NO. Depending on the NO concentration, NO can promote or inhibit carcinogenesis and growth [84,91,92,93].

### 4.1. Cancer-Promoting Role of NO

At low concentrations, NO can promote cancer. The mechanisms of action for this are diverse (see Table 2). At low concentrations, NO can phosphorylate extracellular signal-regulated kinase (ERK) and protein kinase B (AKT) and stabilize hypoxia-inducible factor 1-alpha (HIF1a) [91]. Phosphorylated ERK and AKT induce cell proliferation and inhibit apoptosis. Stabilized HIF1a induces secretion of VEGF, which stimulates angiogenesis. Furthermore, inactivation of caspases 1–4 and 6–8 due to S-nitrosylation by NO inhibits apoptosis [94]. NO inactivates tumor-suppressor p53 protein by inducing GC to AT mutations [79,95]. Other anti-apoptotic effects of NO include inhibition of cytochrome C release, increased expression of BCL-2 to regulate the mitochondrial permeability transition pore [79,95], activation of Cox-2 [95,96], induction of heat shock protein 70 (Hsp 70) and heat shock protein 23 (Hsp 32), and inhibition of ceramide production [95,97].

### 4.2. Cancer-Inhibiting Role of NO

At high concentrations, NO has anti-cancer effects; functions and mechanisms are summarized in Table 3. Most importantly, high concentration of NO upregulates tumor-suppressor p53 protein [101]. p53 blocks the G1/S transition in the cell cycle, which results in apoptosis [102] through the BCL-2 regulated pathway. In BCL-2-regulated apoptosis, cell death is initiated by upregulation of BH3-only proteins induced by p53. BH-3-only proteins bind and inhibit BCL-2 proteins, releasing BAX and BAK, which are apoptotic effectors. Release of BAX/BAK causes mitochondrial outer membrane permeabilization (MOMP) and release of cytochrome C, resulting in cell lysis [103,104]. NO also induces proteosomal degradation of apoptotic proteins. This leads to apoptosis of cancer cells [105]. Anti-cancer activity includes destruction of malignant tumors by inhibition of platelet aggregation through a cGMP-dependent mechanism [97]. NO induces expression of DNA-dependent protein kinases (DNA-PKcs) involved in DNA repair, providing protection against nitrosative and oxidative stress [106]. Furthermore, NO-mediated inhibition of the NFκB /Snail/Yin Yang 1 (YY1)/Raf-1 kinase inhibitor protein (RKIP) circuitry suppresses cancer cell resistance and metastasis [107].

### 4.3. NO and Immune Cells within Cancer Tissues

As mentioned above, a combination of cell types is present in cancer tissue, which is referred to as the tumor microenvironment (TME). The TME is composed of immune cells and nonimmune cells, stromal components, and vasculature [109]. Immune cells in the TME play critical roles in regulating cancer growth and progression [98]. Among the immune cells in the TME, tumor-associated macrophages (TAMs) have pleiotropic functions including antigen presentation and target cell killing. They also remove cell debris, promote tissue remodeling, and regulate inflammation [110]. TAMs display opposite phenotypes depending on the TME in which they are contained. Activated TAMs have an M1 or M2 phenotype. M1 macrophages promote an inflammatory response in response to invading pathogens and cancer cells, whereas M2 macrophages exert an immunosuppressive phenotype to aid tissue repair and cancer progression. M1 macrophages secrete pro-inflammatory cytokines such as interleukin 12 (IL-12), TNF-α, and IFN-γ and express high levels of iNOS. In contrast, M2 macrophages produce anti-inflammatory cytokines such as interleukin 10 (IL-10), 14, and 4 (IL14, IL4) [111]. Macrophages that encounter tumor cells are activated to polarize them to the M1 phenotype, characterized by iNOS induction and simultaneous production of large amounts of NO. High concentrations of NO cause cancer cell death [110]. Furthermore, NO promotes increasing polarization of M1 macrophages in the TME, resulting in NO production through upregulation of iNOS. However, hypoxic TME suppresses iNOS expression, and M2 macrophage-induced anti-inflammatory mediators either neutralize the activity of M1 macrophages or induce repolarization of M1 macrophages to M2 macrophages. Therefore, finding a way to induce M1 macrophages might be an effective method to increase cancer cell death [84]. Recent studies have indicated that glycyrrhetinic acid (GA), the hydrophobic part of GL, induces polarization of macrophages into the M1 phenotype. In addition, GA has been shown to convert M2 macrophages into M1 macrophages [112].

### 4.4. Nitric Oxide and Multidrug Resistance

**Figure 3:**
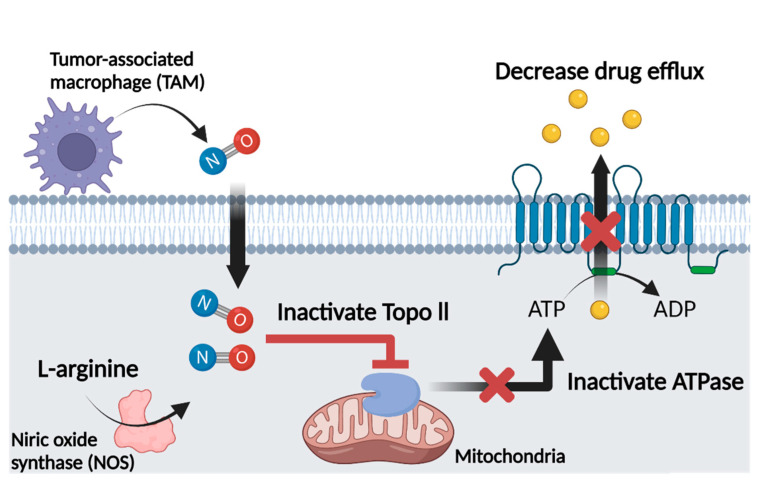
Mechanisms of NO-mediated MDR inhibition. Image created with BioRender icons. NO is produced by TAM or the cancer cell itself, and internalized NO mediates enzyme activities in cells. In mitochondria, NO inactivates topo II enzyme activity by S-nitrosylation. Since topo II is responsible for ATPase activity of ABC transporter, NO-induced topo II inactivation leads to inhibition of ABC transporter. Thus, ABC transporter-mediated drug efflux is decreased, resulting in drug accumulation in the cell.

In recent studies, NO was shown to affect the MDR of cancer cells. Studies have shown that NO can be used to overcome MDR, and the underlying mechanisms are summarized in Figure 3. As mentioned above, ABC proteins such as P-gp and BCRP are ATP-dependent efflux proteins that function as the major transporters of drugs out of cancer cells. Overexpression of ABC transporters has been detected in various cancer types: breast, lung, colon, pancreatic, liver, kidney, and adrenocortical tumors in addition to leukemias, lymphomas, and neuroblastomas. Overexpression is associated with reduced survival rates. Thus, inhibition of the overexpression of ABC transporters would be beneficial in overcoming drug resistance in ABC-overexpressing cancer types [13]. To determine the correlation between NO, ABC transporters, and MDR, it is necessary to review the relationship between NO and topoisomerases. Topoisomerases (topo) are nuclear enzymes that maintain the topology of DNA and many functions of DNA in the cell [13]. There are two isoforms of topoisomerase, topo I and II. Both contain multiple cysteines, and modification of the free SH group in topo II has been shown to decrease its catalytic activity [113]. As mentioned above, NO and its derivatives (RNS) can modify cysteine residues of intracellular proteins by S-nitrosylation. As topo I and topo II contain many reactive SH groups that react with NO generated in the cell, S-nitrosylation by NO occurs, resulting in loss of activity [114]. S-nitrosylation of topo II inhibits its functions, including ATPase activity [18]. Since the ABC transporter is an ATP-dependent protein, ATPase activity decreases when S-nitrosylation occurs at topo II, which affects the activity of the ABC transporter proteins [13,18,114]. Several studies have demonstrated the MDR inhibitory effect of NO. Sinha et al. reported that NO and RNS significantly enhanced adriamycin (ADR) accumulation in P-gp-overexpressing cancer cells. In this study, inhibition of the ATPase activity of P-gp, one of the ABC proteins, by NO and RNS in NCl/ADR-RES cells, which are ovarian cancer cells, decreased drug resistance and increased drug accumulation [13]. Riganti et al. found that NO inhibited doxorubicin efflux and increased its accumulation in P-gp overexpressing HT29-dx cells, which are doxorubicin-resistant human colon cancer cells. These researchers incubated HT29-dx cells with a mix of pro-inflammatory cytokines (such as IFN-γ, TNF-α, and IL-1β) to increase the activity of iNOS to promote NO synthesis. Upregulated iNOS activity and NO concentration increased intracellular accumulation of doxorubicin in HT29-dx cells. They also observed that NO increased drug accumulation in A549 human lung cancer cells and K562 human leukemia cells [115]. Sinha and colleagues also used JS-K as a cancer-specific NO donor. They showed that JS-K was effective at reversing drug resistance in NCl/ADR-RES cells. When they examined the effect of another NO donor, DETNO, in BCRP-overexpressing MCF-7/MX human breast cancer cells, they found that NO from the NO donor directly inhibited the ATPase activity of BCRP, inducing significant intracellular drug accumulation. Interestingly, the MCF-7/MX cell line was resistant to JS-K, suggesting it as a substrate for BCRP. In contrast, DETNO was not a substrate for BCRP and reversed drug resistance in MCF-7/MX cells [116]. However, some studies indicated that NO could induce drug resistance in several cancer cells instead of inhibiting MDR. NO inhibits some topoisomerase active cancer therapeutics activity [114,117]. Mason et al. tested several topo active drugs to various cell lines. They revealed that NO could inhibit activity of etoposide (VP-16) [117]. The interaction VP-16 with topo II leads to cancer cell death by inhibiting DNA repair function of topo II. However, NO produced by iNOS of macrophages in TME reacted with VP-16 leading to decrease of cancer’s cytotoxic effect [117]. They also researched another topoisomerase active cancer drug. Camptothecin (CPT) is a topo I poison, usually used as treatment of cancers in the clinic. They indicated that NO induces wild-type p53 protein (wtp53 protein) and this induces BCL-2 protein stabilization in MCF-7 cancer cell. NO induced downregulated topo I binding to stabilized BCL-2 protein, which could lead to MCF-7 cells becoming highly resistant to CPT [114]. Therefore, it is important to select appropriate NO donors for specific ABC transporter types to reverse MDR. Furthermore, the correlation between NO and cancer cell type and the correlation between anticancer drugs and NO are equally important. In that context we will introduce GL as an NO modulator. Before that, traditional NO donors are summarized below.

### 4.5. NO-Donors

As mentioned above, NO is a short-lived free radical with high reactivity [82]. For this reason, NO does not accumulate in tissues, resulting in limited therapeutic efficacy and side-effects at low concentrations [118]. It is important to ensure high concentrations of NO at specific cancer sites for it to exert a therapeutic effect. Researchers have developed several NO donors for this purpose, as shown in Table 4. There are several classes of NO donors including organic nitrates, N-diazeniumdiolates (NONOates), S-nitrosothiols (RSNO), nitrobenzenes, furoxans, and metal nitrosyl compounds [118,119]. Organic nitrates are the most widely used NO donors. These drugs can be metabolized by specific enzymes such as mitochondrial aldehyde dehydrogenase (mtADH) [119]. NONOates comprise a diolate group bound to electrophilic adducts (primary and secondary amines). A NONOate can produce two NO molecules, and the release of NO occurs spontaneously and does not require metabolism. S-nitrosothiols (RNSO), in contrast to NONOates, produce NO under specific conditions. These compounds have a single chemical bond between a thiol group and the NO. Therefore, S-NO bond cleavage promotes release of NO [118,119]. Nitrobenzene and metal nitrosyl compounds produce NO in response to light irradiation, but the metabolism of these two compounds is quite different. Nitrobenzene’s nitro groups are converted into nitroso groups by light irradiation. Cleavage of the oxygen and nitrogen bonds in the nitroso group results in production of NO. When metal nitrosyl compounds are irradiated with light, photoelectron release from the π orbital of the metal ion to the π antibonding orbital of NO is promoted. As a result, NO is released rapidly from metal nitrosyl compounds. Furoxans are stable under acidic and basic conditions and can produce NO in response to exposure to sulfhydryl compounds such as cysteine [118]. As mentioned above, it is important to select an appropriate NO donor for a specific ABC transporter type to reverse MDR. However, we argue that it would be more efficient to enhance the activity of iNOS using a compound such as glycyrrhizin, a licorice extract, rather than an NO donor.

## 5. Glycyrrhizin as an Anti-Cancer Therapeutic Agent and Nitric Oxide Regulator

**Figure 4:**
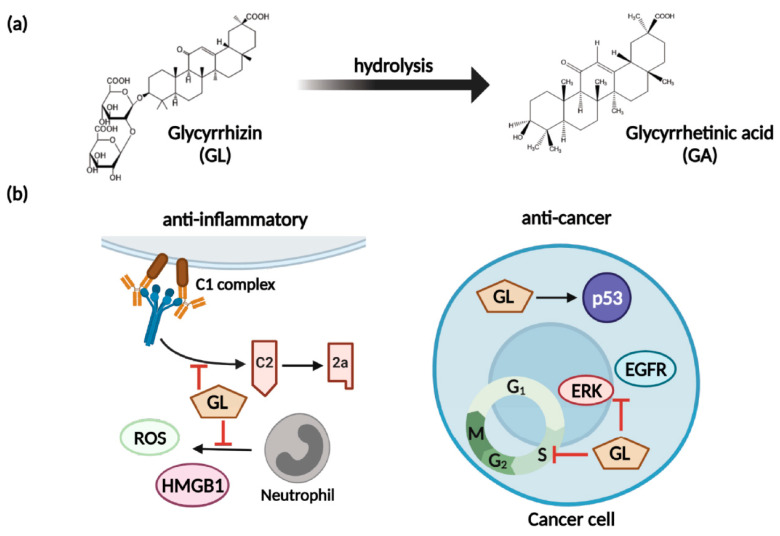
GL metabolism and activities. Image created with BioRender icons. (**a**) GL can be hydrolyzed to GA. (**b**) GL inhibits the cell cycle and ERK and EGFR phosphorylation and activates p53; GL inhibits the C2 pathway and production of ROS and inflammatory cytokines.

GL is an active compound of licorice root and is composed of two molecules of glucuronic acid and one molecule of glycyrrhetinic acid (GA) [125]. GL has anti-inflammatory, anti-viral, anti-microbial, anti-oxidative, and anti-cancer activities (shown in Figure 4) as well as immunomodulatory, cardioprotective, and hepatoprotective effects [126]. To briefly introduce the anti-inflammatory effect of GL, GL inhibited both classical and alternative complement pathways. GL blocks C5 and IA in the complement cascade, in addition to inhibiting the lytic pathway of the complement system [127,128]. GL also inhibits the production of reactive oxygen species (ROS) in neutrophils [126,129]. The anti-inflammatory effect of GL related to ROS was achieved by inhibiting ROS production by neutrophils without ROS scavenging activity [129]. GL inhibits the metabolism of glucocorticoids, which are anti-inflammatory steroids [130,131]. GA, the result of GL hydrolysis, inhibits activation of 11 beta-hydroxysteroid dehydrogenase (11 beta-HSD), which converts hydrocortisone to cortisone. The hydrocortisone is a type of glucocorticoid; inhibiting its metabolism can facilitate its anti-inflammatory effects [130,131].

GL has several anti-cancer-related pharmacological activities such as broad anticancer ability, enhancement of drug absorption by cancer cells, and inhibition of MDR [125]. GL inhibits the migration and invasion of several cancer types and strengthens the immune system [125,132]. GL blocks the protein kinase B/mammalian target of rapamycin/signal transducer and activator of transcription 3 pathway (AKT/mTOR/STAT3 pathway), downstream factors, and cyclin D1 and reduces survival [133]. The reduction in pro-proliferative factors induces cell cycle arrest in the G0/G1 phase, inhibiting cell proliferation and preventing cells from entering the G2 phase [134]. GL induces activation of tumor-suppressor p53 [135] and inhibits phosphorylation of mitogen-activated protein kinase (MAPK), ERK, and epidermal growth factor receptor (EGFR), inhibiting cancer cell metastasis and proliferation, leading to apoptosis and anti-angiogenesis [132,136]. Several studies have shown that GL can act as an NO regulator [16,112], and it inhibits the development of MDR in cancer cells [19,20]. In addition, GL itself can inhibit ABC transporter activity [137,138].

### 5.1. Glycyrrhizin and Nitric Oxide

**Figure 5:**
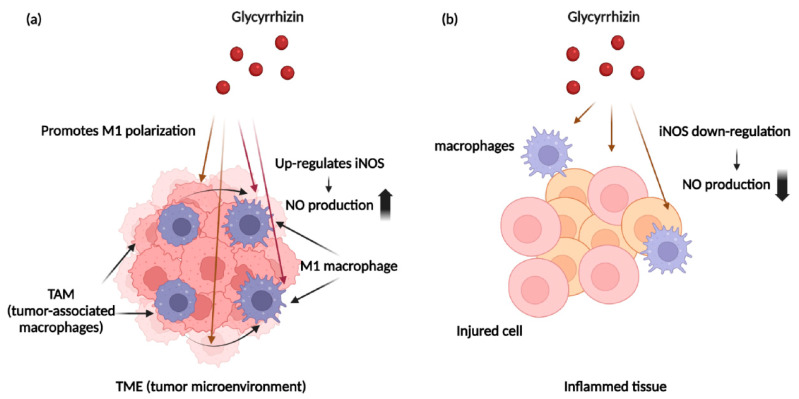
Mechanisms by which GL acts as an NO regulator. Image created with BioRender icons. (**a**) GL induces polarization of TAMs to the M1 phenotype and upregulates iNOS expression of M1 TAMs, resulting in increased NO production; (**b**) NO acts as pro-inflammatory cytokine and induces inflammation in inflammatory diseases. GL downregulates NO production via reducing iNOS expression of cells of inflamed tissue.

As mentioned above, NO can promote or inhibit carcinogenesis and growth depending on its concentration. To determine the association between NO and GL, we focus on the relationship between tumor-associated macrophages (TAMs) and GL. As mentioned above, M1 macrophages can increase NO production by cancer cells and macrophages. Several studies have shown that GL can induce macrophages to produce NO by polarizing macrophages to the M1 phenotype and upregulating iNOS [16,112]. Hong et al. reported that GL enhanced nitric oxide production in IFN-γ-activated macrophages, and that upregulated NO resulted in cancer cell death by stimulating the secretion of pro-inflammatory cytokines [16]. Kondo et al. found that GL-induced peritoneal macrophages produce enhanced amounts of NO. They found that GL-induced macrophages produce three times the amount of NO compared to saline-induced macrophages in response to LPS for 48 h. However, they have not figured out the mechanism for this [139]. Yulong et al. showed that GA, the hydrophobic part of GL, promoted macrophage polarization to the M1 phenotype. They confirmed M1 macrophage polarization by demonstrating increased expression of the M1 macrophage-related marker C-C chemokine receptor type 7 (CCR7) in addition to TNF-α, IL12, and IL-6, and decreased expression of the M2 macrophage-related markers mannose receptor (MR) and chitinase-like protein (Ym1). They stated that GA induces activation of NF-κB and JNK in macrophages, inducing polarization into the M1 phenotype. They also showed that GA inhibited the phosphorylation of ERK1/2, which led to increase in NO and pro-inflammatory cytokine production [112]. Another study investigated the effect of NF-κB as an NO upregulator enhanced by GL. Jeong et al. reported that GA upregulated iNOS expression in macrophages though dose-dependent NF-κB transactivation. The increased NF-κB level led to an increase in iNOS expression and increased production of NO. The correlation between GL, NF-κB, and iNOS was double-checked through comparison of NO production between an NF-κB inhibitor-treated group and GA-treated group [17]. Based on the findings of the studies outlined above, GL induces the polarization of macrophages to the M1 phenotype and increases the production of NO by increasing the level of NF-κB in activated M1 macrophages [16,17,112]. Glycyrrhizin has an anti-cancer effect and stimulates macrophages to increase NO production. Unlike NO donor drugs, GL does not deliver NO directly but induces NO production through iNOS, making it unnecessary to select specific NO donors to block specific ABC transporters. In addition, because GL regulates the iNOS activity of M1 macrophages in a dose-dependent manner, it can reduce the potential side-effects of NO [17]. However, NO upregulation in TME can lead to the effect of lowering MDR, whereas NO upregulation in other tissues can lead to promotion of inflammatory action. GL has been shown to enhance NO production by inducing TAM polarization in TME, but reversely inhibit NO production in other tissues. Several studies have shown that GL acts as an NO regulator in inflammatory diseases. R. Tanemoto et al. induced inflammation in rat primary hepatocytes by treatment with IL-1β. Thereafter, the amount of NO reduction was observed by the GL treatment. As a result, GL was shown to inhibit NO production in the hepatocytes of IL-1β-treated mice [140]. Lee et al. revealed the effect of GL on an acute lung injury (ALI) mouse model. They used LPS to induce an ALI model, and iNOS expression was upregulated in the LPS-induced ALI model. GL was shown to lower iNOS levels upregulated due to inflammation to control levels [141]. Wang et al. induced paw edema to mice by treating 1% carrageenan to right hind paw. GL delivered by intraperitoneal injection downregulates iNOS level upregulated due to inflammation by edema [142]. Collectively, GL acts as NO inducer in TME and as NO reducer in other inflammatory diseases. Figure 5 shows a brief diagram of these roles of GL. This suggests that GL acts as an NO regulator.

### 5.2. Glycyrrhizin and Multidrug Resistance

**Figure 6:**
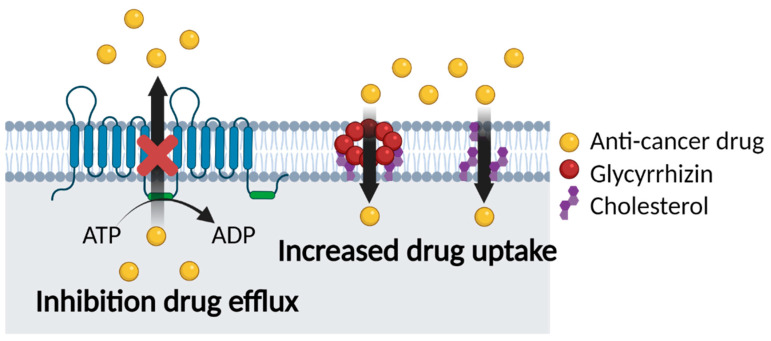
Mechanisms by which GL exerts anti-MDR activity. Image created with BioRender icons. Mechanisms include inhibition of ABC transporters (e.g., P-gp, MRP); GL is incorporated into the lipid bilayer and binds to cholesterol to form pores; GL modifies cell membrane components, thereby increasing elasticity and permeability.

Many studies of chemotherapy have found that GL increases the efficacy of drugs. This is due to the ability of GL to modify cell membrane lipids, including cholesterol, which is involved in membrane elasticity and permeability. GL not only affects cholesterol biosynthesis, but also increases spin–spin relaxation through interaction with cholesterol. Moreover, the interaction of GL with cholesterol results in pore formation in the cell membrane, which increases elasticity and permeability and therefore drug absorption [19,20,125,143]. This suggests that GL has a potential role in reducing MDR by inhibiting a decrease in drug uptake. Furthermore, mechanisms by which GL inhibits MDR are summarized in Figure 6.

#### 5.2.1. Co-Delivery of GL and Anti-Cancer Drugs

Furthermore, GL has MDR-reducing effects through P-gp inhibition, so many attempts have been made to use GL as a P-gp inhibitor in chemotherapy. These in vitro and in vivo attempts are briefly presented in Table 5. Because of overexpression of P-gp in the colon, chemotherapies for colon cancer suffer from the development of MDR. Aconitine, extracted from plants in the genus *Aconitum*, has anti-cancer effects that have been exploited for colon cancer treatment. L. Chen et al. used GL as a P-gp inhibitor and aconitine as an anti-cancer drug for colon cancer in vivo [137]. Co-delivery of GL and aconitine to the intestine significantly increased the plasma concentration of aconitine 1.63-fold compared to delivery of aconitine alone. This absorption enhancement due to P-gp inhibition increases the bioavailability of aconitine [137,138]. Zhou and Wink investigated the effects of three plant extracts on MDR, including GL. Combination treatment of human colon cancer cells (Caco-2) with GL and DOX enhanced the cytotoxicity of DOX. Reduction in the MDR effect was attributed to P-gp inhibition by GL [144]. Cisplatin is used for hepatocellular carcinoma treatment but has poor therapeutic efficacy due to MDR. Wakamatsu et al. attempted to overcome this problem through co-delivery of GL and cisplatin. GL increased cisplatin accumulation in hepatocellular carcinoma cells from 36.4% to 47.7% and functioned as a competitive inhibitor of MRP2 and MRP3, resulting in a reduction in MDR [145]. One suggested mechanism of P-gp inhibition by GL is inhibition of the ATPase activity of P-gp. Co-treatment of P-gp overexpressing human carcinoma cells (KB-C2) with GL and DOX revealed that GL significantly enhanced DOX cytotoxicity relative to DOX treatment alone because GL inhibited verapamil-stimulated P-gp ATPase activity [146].

#### 5.2.2. GL Carriers for Cancer Therapy

In cancer chemotherapy, carriers are used as effective tools for delivering anti-cancer agents to cancer cells. In this regard, studies using GL as carriers for anti-cancer drugs have been actively conducted. There are several advantages using GL as carriers: GL has anti-cancer effects itself, anti-MDR effect through increasing cell membrane elasticity and permeability, and enhancement of cellular internalization due to its negative charge [125,140,150]. Paclitaxel is widely used as an anti-cancer drug, but it has poor oral bioavailability due to P-gp-mediated MDR in intestine [151]. Therefore, Fu-Heng Yang et al. used GL as micelle carrier to improve oral bioavailability of paclitaxel. Paclitaxel-loaded GL micelles showed significant enhancement absorption in jejunum and colon intestine, resulting in 6-fold higher blood concentration [152]. Qihong Zhang et al. also used GL as carrier to deliver camptothecin. Camptothecin is an anti-tumor drug that has a lack of stability and solubility. Qihong Zhang and researchers made amptothecin micelles self-assembled from GL and tannic acid, and drug-loaded micelles show stability and permeability in vitro. Furthermore, drug-loaded micelles enhance anti-cancer effect more than amptothecin alone [153].

## 6. Role of Glycyrrhizin as a Nitric Oxide Regulator in Cancer Chemotherapy

Because NO is highly reactive, it is converted easily into RNS, leading to activation or inactivation of proteins and anti-cancer or pro-cancer effects. Several studies have shown that this effect is regulated mainly by the concentration of NO as described in detail above. A high concentration of NO activates p53, a tumor suppressor protein, and causes apoptosis. Furthermore, a high concentration of NO inhibits topo2 activity by S-nitrosylation. Reduced topo2 activity decreases ATPase activity, which in turn decreases the activity of ATP-dependent ABC transporters and inhibits MDR in cancer cells. NO donors have been used to deliver high concentrations of NO to target tissue, but appropriate NO donors for the type of cancer must be chosen because some NO donors are substrates for specific ABC transporters. As an alternative, we propose using GL, an iNOS inducer, to increase the NO concentration in the TME. 

**Figure 7:**
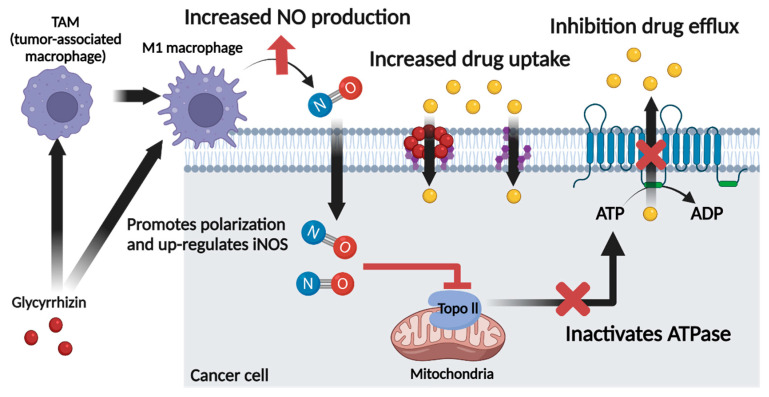
Mechanisms by which GL acts as an NO regulator and MDR inhibitor. Image created with BioRender icons. GL induces polarization of TAMs to the M1 phenotype and upregulates iNOS expression, resulting in increased NO production; GL also inhibits MDR directly through increased drug uptake and inactivation of ATPase.

GL itself has anti-inflammatory, anti-viral, and anti-cancer activity and exerts MDR inhibitory effects by modifying the lipid composition of the cell membrane or directly inhibiting ABC transporters. In addition, according to several studies, GL increases the production of NO by inducing the polarization of macrophages in the TME into the M1 phenotype, resulting in increased iNOS activity. Although the relationship between the GL-induced increase in NO and inhibition of MDR has not been investigated directly, we expect GL to inhibit MDR by increasing the NO concentration in the TME. Furthermore, GL itself has an MDR inhibitory effect and is expected to enhance drug accumulation in cancer cells when co-delivered with anti-cancer drugs. Therefore, we suggest that GL can act as both an NO regulator and an MDR inhibitor via the mechanisms shown in Figure 7.

## 7. Conclusions

Cancer is a leading cause of death worldwide, and numerous therapies have been developed to treat cancer. Among them, chemotherapy is one of the most effective therapies but suffers from the development of MDR. MDR, or the resistance of cancer cells to chemical drugs, is mediated mainly by drug efflux by ABC transporters present in the cell membrane of cancer cells. NO has emerged as an effective method for inhibiting MDR, and studies have focused on finding effective ways to deliver NO to the tumor microenvironment. However, NO delivery is limited by the risk of potential side effects due to inappropriate concentrations and difficulties in delivery to the appropriate location. Therefore, we propose a method to regulate the production and concentration of NO in the TME using GL rather than direct delivery of NO via NO donors. Multiple studies support the efficacy of GL as an anti-cancer and anti-MDR agent. Additionally, several studies have shown that GL can modulate NO production at appropriate concentrations for NO to have efficacy. We therefore expect GL to enhance the efficacy of cancer chemotherapy as both an NO regulator and direct MDR inhibitor.

## Figures and Tables

**Table 1:** Co-delivery of ABC transporter inhibitors and anti-cancer drugs.

Cancer Cell Type	Anti-Cancer Drug	ABC Transporter	Inhibitor	References
Breast	Doxorubicin	P-gp	PSC-833	[59]
	Doxorubicin	P-gp	Verapamil	[60,61]
	Doxorubicin	P-gp	Cyclosporine A	[62]
	Paclitaxel	P-gp	Elacridar	[63]
	Doxorubicin	BCRP	Lapatinib	[64]
	Doxorubicin	BCRP	Pluronic L61	[65]
	Gefitinib	BCRP	Fumitremorgin C	[66]
	Paclitaxel	BCRP	Sitravatinib	[67]
	Paclitaxel	BCRP	Lapatinib	[68]
Lung	Paclitaxel	P-gp	Pluronic P123/F127	[69]
	Gefitinib	P-gp	Cyclosporine A	[70]
Ovarian	Paclitaxel	P-gp	Tariquidar	[71]
	Paclitaxel	P-gp, MRP1	Curcumin	[72]
Brain	Vincristine	MRP1	Reversan	[73]
	PF-2545920	MRP1	Reversan	[74]

**Table 2:** Cancer-promoting physiological function of low concentrations of NO.

Physiological Function	Mechanisms of Action	References
Cell proliferation	Phosphorylation of proteins in the ERK and AKT pathways	[91,97]
	Phosphorylation of proteins in the JNK/SAPK pathway	
	Phosphorylation of proteins in the P38K pathway	
Anti-apoptotic effect	Inactivation of caspases 1–4 and 6–8	[79,95,97]
	Inactivates p53 protein by inducing GC to AT mutation	
	Activation of cyclooxygenase-2	
	Induction of Hsp 70 and Hsp 32	
	Inhibition of ceramide production	
Cell survival	PI3K/AKT pathway upregulation	[83,91,97,98]
	β-Catenin downregulation	
Angiogenesis	Stabilization of HIF1a	[91,92,93,95,97,99]
	Induction of VEGF secretion	
	Downregulation of Angiostatin and Thrombospondin-1	
	Induction of interleukin-8 secretion	
Migration and invasion	Upregulation of α2β1 integrin	[97,100]
	Upregulation of matrix metalloproteinases (MMPs)	

**Table 3:** Cancer-inhibiting physiological function of high concentration of NO.

Physiological Function	Mechanisms of Action	References
Apoptosis	Upregulation of tumor-suppressor p53 protein	[101,102,103,104,105]
	Induction of the BCL-2 regulated apoptotic pathway	
	Release of cytochrome C	
	Induction of proteosomal degradation of anti-apoptotic proteins	
Anti-invasion and Anti-metastatic effects	Downregulation of E-selectine	[97,107,108]
	Inhibition of platelet aggregation	
	Inhibition of the NF-κB/Snail/YY1/Raf-1 kinase inhibitor protein (RKIP) circuitry	

**Table 4:** Classes of NO donors and NO-producing mechanisms.

Class of NO Donor	Type	NO-Producing Mechanism	References
Organic nitrates	Glyceryl trinitrate (nitroglycerin) Isosorbide dinitrate Nicorandi Nipradilol Pentaerythrityl tetranitrate	Can be metabolized by specific enzymes such as mitochondrial aldehyde dehydrogenase (mtADH)	[118,119,120]
N-Diazeniumdiolates (NONOates)	PYPRO/NO DEA/NO DETA/NO PROLI/NO JS-K	Produces two NO molecules; release of NO occurs spontaneously and does not require specific metabolism	[118,119,120]
S-Nitrosothiols (RSNO)	S-nitrosoglutathione S-nitroso-N-acetylpenicillamine S-nitroso-N-valerylpenicillamine	S-NO bond cleavage promotes release of NO	[118,119,120]
Nitrobenzenes	PhNO_2_ -1 PhNO_2_ -2	Nitro groups are converted into nitroso groups by light irradiation, leading to cleavage of oxygen and nitrogen bonds to produce NO	[118,120,121]
Furoxans	Furoxans-1 Furoxans-2 Furoxans-3	NO produced in the presence of sulfhydryl compounds	[118,122]
Metal nitrosyl compounds	Ru-NO Fe-NO Sodium nitroprusside dihydrate	Photoelectron release from the π orbital of the metal ion to the π antibonding orbital of NO is promoted by light irradiation. As a result, NO is released rapidly from metal nitrosyl compounds	[118,123,124]

**Table 5:** Co-delivery of GL and anti-cancer drugs.

Cancer Cell Type	Anti-Cancer Drug	Delivery Method	Reference
Colon	Doxorubicin	KB-C2 cells were treated with GL (100 μM) and doxorubicin (5 ng/mL)	[146]
	Aconitine	Oral administration of GL (50 mg/kg) and aconitine (0.2 mg/kg)	[137]
	Asiatic acid	Oral administration of GL (100 mg/kg) and asiatic acid (20 mg/kg)	[138]
Liver	Cisplatin	Hepatocellular carcinoma cells were treated with GL (100 μg/mL) and cisplatin (10 ng/mL)	[145]
	Doxorubicin	Alginate nanogel particle	[147]
		Polymeric prodrug micellar carrier based on polyethylene glycol-derivatized GA	[148]
	Entecavir	Albumin nanoparticle	[149]
